# Multi-chimera states in FitzHugh-Nagumo oscillators

**DOI:** 10.1186/1471-2202-14-S1-P303

**Published:** 2013-07-08

**Authors:** Philipp Hövel, Iryna Omelchenko

**Affiliations:** 1 Institut für Theoretische Physik, Technische Universität Berlin, Berlin, 10623, Germany; 2 Bernstein Center for Computational Neuroscience, Humboldt-Universität zu Berlin, Berlin, 10115, Germany

## 

Chimera states are spatio-temporal patterns of synchrony and disorder observed in homogeneous oscillatory media with nonlocal coupling. They are characterized by coexistence of distinct spatial regions with regular synchronized and irregular incoherent motion. Initially discovered for phase oscillators, chimera states have been also found in systems of nonlocally coupled discrete maps [1], time-continuous chaotic systems [2] and have been recently realized in experiments [3].

We demonstrate the existence of chimera states for neural oscillators [4]. In detail, we investigate the cooperative dynamics of nonlocally coupled neural populations modeled by FitzHugh-Nagumo systems, where each individual system displays oscillatory local dynamics described by a fast activator and slow inhibitor variable. Applying a phase-reduction technique we show that off-diagonal nonlocal coupling is a crucial factor for the appearance of chimera states. In the considered configuration, this off-diagonal coupling is realized by a rotational coupling matrix that mixes input of the activator and inhibitor and that is conveniently described by the coupling phase as a single control parameter. We analyze the stability of chimera states in the parameter space of the system and discuss mechanisms of transitions between different chimera types.

Surprisingly, we find that for increasing coupling strength classical chimera states undergo transitions from one to multiple domains of incoherence. Then patches of synchronized dynamics appear within the incoherent domain giving rise to a multi-chimera state. We find that, depending on the coupling strength and range, different multi-chimeras arise in a transition from classical chimera states. The additional spatial modulation is due to strong coupling interaction and thus cannot be observed in simple phase-oscillator models.
